# The spread of genomics

**DOI:** 10.3389/fgene.2026.1985860

**Published:** 2026-10-21

**Authors:** Andrew H. Paterson, Brock A. Harpur

**Affiliations:** 1 Department of Crop, Soil, and Environmental Sciences, Auburn University, Auburn, AL, United States; 2 State Key Laboratory of Crop Genetics and Germplasm Enhancement and Utilization, Jiangsu Key Laboratory for Horticultural Crop Breeding, College of Horticulture, Nanjing Agricultural University, Nanjing, China; 3 Henan Sesame Research Center, Henan Academy of Agricultural Sciences, Zhengzhou, Henan, China; 4 Department of Entomology, Purdue University, West Lafayette, IN, United States

**Keywords:** Columbian exchange, diversity, DNA marker, genome, honey bee, plant, QTL mapping

## Abstract

Once a very specialized area limited to a few model systems and well-equipped labs, genomics has become routine with sophisticated methods commercially available at manageable cost. Leveraging technologies developed for biomedical applications, plants and plant-associated organisms are experiencing a revolution in the characterization of their genomic ‘hardware’ (genes and genomes), ‘software’ (regulatory cues), and variation therein. Their experimental facility made plants an early model for dissection of complex traits into individual genetic components, initially in designed populations -- however economies of scale permit genome-wide scans in feral or natural populations, exemplified by a plant-associated organism, the honey bee (*Apis mellifera*). Similarly, massively parallel methods enable comprehensive study of genome-wide gene expression, documentation of spectra of genetic variation within and among gene pools, and composition of and interactions among ecosystem constituents. The ability to reach beyond controlled experimental crosses is exemplified by study of ‘Columbian Exchange’ taxa, in which genetic bottlenecks a few hundred generations ago still provide sufficient linkage disequilibrium for genome scans using current methods. Already permeating biomedicine, genomics may have even greater implications that are yet to be fully appreciated across life sciences, for example, documenting and deploying natural variation to improve agricultural productivity and sustainability.

## What changed recently

### Genome sequencing

Once a major undertaking to obtain the sequence of even a single gene (Min Jou et al., 1971), massively-parallel technologies have transitioned whole-genome sequencing from dispersed efforts involving complex coordination among many international teams, to being commercially available at a cost manageable even to most individual public research labs. Currently available genomes sample most major clades but fewer than 0.4% of the ∼400,000 known green plant species (Lughadha et al., 2016), with ongoing progress expected from initiatives such as the Earth BioGenome Project (Lewin et al., 2018) the Darwin Tree of Life (Consortium et al., 2022), and PLAN*e*T (Wang et al., 2026).

An ever-growing slate of both long-standing questions and new opportunities in life sciences research and application have been empowered by access to ever-improving genome assemblies (Keenan et al., 2025), reduced-representation methods that empower acquisition of haplotype information for large numbers of individuals at nominal cost (Andrews et al., 2016), and massive sampling of expressed nucleic acid sequences (Torres-Rodríguez et al., 2025).

### Genetics of complex traits

A long-standing and cross-cutting challenge in agriculture, evolution and medicine has been quantitative inheritance, the principle that many differences between organisms in the same gene pool are due to the collective effects of subtle variations in large numbers of genes. From a whole-phenome perspective, a rich literature exemplified by pioneers such as Wright (1968) has provided insight into quantitative inheritance and the notion that individual ‘quantitative trait loci’ (QTLs) underlying quantitative variation might be identified is more than a century old (Sax, 1923). Seminal work with morphological markers (Thoday, 1961) and isozymes (Tanksley et al., 1982) further enriched the intellectual backdrop for what might be accomplished with genome-wide tools.

Crop plants and their relatives, that could be routinely crossed without ethical issues to form large populations which could be maintained at modest cost and measured for suites of economically important traits, were a natural test bed for the DNA-based whole-genome mapping methods envisioned by pioneers of the Human Genome Project (Botstein et al., 1980). Early examples (Edwards et al., 1987; Paterson et al., 1988) spawned a rich literature of agricultural, ecological and even medical genomics, identifying diagnostic DNA markers that could be assessed independently of phenotype as predictive tools to select for traits, or identify likely disease subjects or carriers and guide early interventions.

An ongoing challenge in the ‘molecular dissection’ of complex traits was the need for populations in strong linkage disequilibrium, i.e., with DNA markers linked to trait-determinants no more than a few centiMorgans away, with detailed genetic maps providing closely-spaced markers suitable for ‘interval mapping’ methods (Lander and Botstein, 1989; Zeng, 1993).

An early example using linkage disequilibrium to establish a relationship between a trait and nearby single-nucleotide polymorphism (Ozaki et al., 2002), spawned a new approach, genome-wide association studies (GWAS), using historical accumulations of recombination as a complement to recent crosses, for example, as detailed by Slatkin (2008). GWAS could thus be used to search for linkages and causal genes in the absence of designed crosses, indeed even in wild populations (Santure and Garant, 2018) or those derived from recent founders within a few hundred generations. Somewhat serendipitously, this window in time is consistent with the ‘Columbian Exchange’, the extensive bidirectional flow of plants, animals and microbes between the Old and New Worlds resulting from human migration.

Taking full advantage of historical recombinations required low-cost methods of accessing vast numbers of closely-spaced DNA polymorphisms. First relying on reduced representation methods (Davey et al., 2011) that are gradually being rendered less economic as whole-genome sequencing costs drop, genomics has been democratized, and throughput dramatically increased. Today, even whole genomes can be sequenced for $100 (https://frontlinegenomics.com/the-100-genome-wheres-the-limit/) and where economical, reduced-representation approaches can take advantage of well-established haplotypes to home in on rare variants.

### Gene expression

A recent review envisions the entire hereditary information of an organism as ‘hardware’, and the associated regulatory cues as ‘software’, noting that the sessile nature and indeterminate development puts strikingly different demands on gene regulation in plants than animals (Paterson and Queitsch, 2024).

While plant gene regulation research lacked the unified, comprehensive and well-funded ENCODE and ModENCODE projects that annotated transcripts, transcription factor binding sites, histone modifications, ACRs, and long-range regulatory interactions, the methods developed and employed in these (ENCODE, 2012; Sanyal et al., 2012; Thurman et al., 2012) and other projects have been adapted for use in plants in many studies. Epigenetic marks reinforce patterns of cell-type and condition-specific gene expression and contribute to appropriate transcriptional responses (Sullivan et al., 2014; Schmitz et al., 2022). Accessible chromatin regions (*i.e.*, regulatory DNA, ACRs) are enriched for trait-associated genetic variation implicated in domestication and evolution (Maurano et al., 2012; Sullivan et al., 2014; Rodgers-Melnick et al., 2016). Although researchers have learned some of the rules that govern the transcription of genomic information in plants, there is much left to be discovered.

In much the same manner that genome scans using reduced representation approaches reveal non-random associations between specific genomic loci and traits, *en masse* sequencing of messenger RNA has been envisioned to provide a basis to seek associations of particular transcripts with traits. Transcriptome-wide association studies, both in animals (Gusev et al., 2016) and plants (Hirsch et al., 2014), have become a useful element of both transmission and developmental genetic studies, to complement positional genetics toward identifying pathways and specific genes implicated in trait variation (Torres-Rodríguez et al., 2025).

### Variation within populations

Naturally-occurring alleles, ‘biased’ in informative ways by the action of selection, provide both a useful tool to infer gene function, and a valuable data set for genetic improvement applications. The value of diverse germplasm for GWAS, above, often draws upon large germplasm collections such as many collected and phenotyped for numerous crops by CGIAR centers, revealing much of the spectrum of naturally occurring alleles in a gene pool, while also evaluating associations of the more abundant alleles with traits [e.g., (Morris et al., 2013)]. In addition to *de novo* searches for trait association, phenotypic and passport information for such collections can be of value in testing hypotheses, for example, regarding the geographic distribution of specific candidate alleles (Cuevas et al., 2016).

### Complexity of associations between taxa

Higher organisms have evolved in a world long- and numerically-dominated by microorganisms, and comprise ecosystems of host and associated microbial cells (the ‘microbiome’) working in concert to produce functionality that was previously attributed to the host alone. Metagenomics and associated approaches have increased appreciation that ‘individual higher organisms’ are actually ecosystems of host and associated microbial cells (the ‘microbiome’) working in concert to produce functionality that was previously attributed to the host alone (and reaching far beyond well-studied cases such as nitrogen-fixing legumes) (Rout and Southworth, 2013; Rout, 2014).

The genetic diversity present in many microbiomes can resemble or even exceed that of their hosts. For example, microbial cells in and on humans are similar in number to the human cells (Sender et al., 2016). It follows that intimate and complex relationships between multicellular hosts and their associated unicellular organisms have evolved; and that these span the range from parasitic to mutualistic (Friesen et al., 2011). Sessile organisms such as plants may be even more intricately intertwined with their (micro) biotic environments than motile organisms, and the literature is replete with examples of microbially-mediated plant growth promoting (PGP) activities; e.g., nitrogen (N_2_)-fixation, phosphate solubilization, production of indole-3-acetic acid (IAA), ACC deaminase, and phytohormones (Glick et al., 2007).

## Case study of a plant-associated organism: the honey bee in North America

Consistent with the integrative approach adopted by the “Plants and Plant-associated organisms” section and the cross-taxon applicability of genomics tools, an example of how these advances have impacted a non-model taxon derive from the Western honey bee, *Apis mellifera*. Beyond its tremendous importance to the world food supply, the honey bee is well-suited as a challenging example in that: (a) it had been absent from the New World for nearly 10 million years, between the extinction of a relative known only from fossils and the post-Columbian introduction of Old World strains which have spread across the continent; (b) it is genetically complex, colonies having one maternal parent and typically a dozen paternal parents (polyandry); and (c) it has unusual population structure, with very few reproductive individuals and haplodiploidy, male ‘drones’ being derived from unfertilized eggs thus representing only their maternal genotype and only a subset thereof.

The New World is not part of the native range of extant honey bees (genus *Apis*). Although *Apis* was present in North America during the Miocene, it went extinct on the continent long before human arrival (Engel et al., 2009). The genus was reintroduced to North America only in the early colonial period when *A. m. mellifera* was carried to the eastern seaboard by European settlers around or before 1620 (Carpenter and Harpur, 2021). Over the following two centuries, deliberate introductions of additional lineages (most consequentially the Italian *A. m. ligustica*) layered additional genetic contributions onto this founding population, so that the contemporary North American population is a human-mediated admixture whose diversity can exceed that of any single ancestral source (Sheppard, 1989; Harpur et al., 2012; Cridland et al., 2018). This admixed, historically documented population is what makes the North American honey bee a tractable case study. Its demographic history is comparatively well constrained, and its genome has now been interrogated at essentially every scale of resolution.

### Waves of diversity

The genetic architecture of the North American honey bee is best understood as a stratigraphy of introductions. Native *A. mellifera* diversity is partitioned into deeply divergent evolutionary lineages classically called the western European (M), eastern European (C), African (A), and Middle Eastern (O) groups first delimited on morphometric grounds (Ruttner, 1988) and later resolved genomically (Whitfield et al., 2006; Dogantzis et al., 2021). The colonially-introduced lineage (M; *A. m. mellifera*) was overlaid by nineteenth-century imports of C-lineage bees (*A. m. ligustica*, *A. m. carnica*), while the escape of A-lineage *A. m. scutellata* in Brazil began the “Africanized” expansion that reshaped the genetic landscape of the southern United States (Carpenter and Harpur, 2021; Whitfield et al., 2006; Nelson et al., 2017; Calfee et al., 2020). Because management repeatedly mixes these lineages, human-managed populations can carry more standing variation than any single wild source (Harpur et al., 2012). The finer demographic detail of these introductions, including the surprising rapidity with which local composition can turn over, has since been recovered directly from historical and museum material (Cridland et al., 2017; Cridland et al., 2018), and even the deep origin of the species has been revised from an African to a proposed Asian center (Dogantzis et al., 2021).

### Genetic mapping

Genetic mapping in honey bees has always been constrained by their reproductive biology. Queens mate on the wing with many drones, so controlled crosses are impossible in the field and depend on instrumental insemination, a specialized surgical technique (Laidlaw, 1944). Even with it, the sample sizes required can exceed what a single apiary can maintain (Fikere et al., 2026). Early linkage work tended to rely on a handful of visible morphological mutants and produced only sparse maps. The first genome-wide map arrived with the RAPD-marker map of Hunt and Page (Hunt and Page, 1995), which identified 26 linkage groups and initially revealed the honey bee’s exceptionally high recombination rate. Subsequent work confirmed it to be roughly an order of magnitude above the metazoan average (Beye et al., 2006). Subsequent microsatellite linkage maps (Solignac et al., 2007) anchored the genetic map to the physical genome, and once the reference assembly was available (next section), mapping shifted onto a genome-wide, SNP-based approach.

### Reference genome sequencing

The honey bee was among the earliest agricultural species sequenced, the first Hymenopteran and fourth insect. Its genome proved to be unusually AT-rich, comparatively slowly evolving, and notably depleted in transposable elements relative to *Drosophila* (Consortium, 2006). That first assembly (Amel_4.0) was also fragmented, and its extreme base composition and long AT-rich intergenic tracts frustrated both assembly and gene prediction. A community-led upgrade combined ‘new’ short-read and Roche 454 data with improved annotation (Amel_4.5) recovered thousands of previously missing or mismodeled genes (Elsik et al., 2014). Long-read and long-range scaffolding technologies yielded a chromosome-length assembly (Amel_HAv3.1) that closed most remaining gaps and resolved many repetitive and GC-heterogeneous regions that had systematically eluded earlier drafts (Wallberg et al., 2019). As the rest of this issue will surely highlight, reference genome quality is a moving standard.

### Genomic diversity

Characterizing honey bee diversity in North America is a history of marker technology. Early allozyme surveys were low-resolution, partly because Hymenoptera show unusually low allozyme heterozygosity (Metcalf et al., 1975). Mitochondrial COI–COII regions helped resolve the deep A, M, C, and O lineages genetically and identify their presence in the continent (Garnery et al., 1992; Franck et al., 2000). Because mtDNA follows only the maternal line, it was complemented from the 1990s by nuclear microsatellites (Estoup et al., 1995; Franck et al., 1998; Franck et al., 2001). The reference genome then moved the field from a handful of loci to genome-wide resolution. Whole-genome resequencing dated lineage splits and showed diversity to be high and shaped by demographic history rather than domestication bottlenecks (Wallberg et al., 2014), North American sampling quantified local diversity (Harpur et al., 2012; Harpur et al., 2014; Cridland et al., 2017; Cridland et al., 2018; Calfee et al., 2020).

### Linking DNA diversity to traits

Connecting variation to mechanism began, for honey bees, in the pre-genomic era and continues now in the post-genomic era. Social immunity, or hygienic behavior, was the first behavioral trait identified as having a genetic basis. Hygienic behavior is the removal of diseased brood by nurse bees, and was among the first animal social behaviors genetically dissected (Rothenbuhler, 1964). Since then, QTL mapping has refined the regions associated with the trait (Lapidge et al., 2002; OXLEY et al., 2010). More modern genomic approaches have opened up honey bees to further genetic dissection. The genomic basis of artificially selected high-royal-jelly strain (Cao et al., 2021), signatures of climatic and thermal adaptation in European and desert-dwelling subspecies (Henriques et al., 2018; Alghamdi and Alattal, 2023), in the “thelytokous” clone of *A. mellifera capensis*, a single locus governing the parasitic laying-worker phenotype (Lattorff et al., 2007; Aumer et al., 2019), and most recently spontaneous eye-colour mutations (Shultz et al., 2026) Most translationally urgent is resistance to *Varroa destructor*, where mapping of *Varroa*-sensitive hygiene is beginning to yield candidate loci and marker-assisted selection tools (Spötter et al., 2016; Guarna et al., 2017; Conlon et al., 2019). Building on decades of pedigree-based breeding-value estimation (Bienefeld et al., 2007; Brascamp and Bijma, 2014), the field has now moved to genomic prediction (Bernstein et al., 2023).

## What still blocks progress

Arguably one of the greatest barriers to progress in deeper understanding and more effective management of plants and plant-associated organisms, particularly Columbian Exchange taxa, is that their distributions are often now international. The biology of an organism is shaped by its environment and therefore reflects the path by which it has dispersed, often aided by humans either intentionally or not-but multiple barriers hinder study of changes along the path. Broad and deep sampling-species, local populations, breeding lineages -- is sometimes blocked across geographic boundaries (that the organisms themselves regularly cross) by both practical and legal constraints.

There is natural resistance, and for good reason, to allow international exchange of living organisms known to be invasive or actively harmful-notwithstanding that the organisms themselves do not recognize national boundaries. Consequences of such actions can be remarkably rapid; for example, the intentional import to Brazil of *A. mellifera* race scutellata, followed by its rapid escape and spread over ∼50 years; or the introduction of *Sorghum halepense* to the southeastern United States shortly after the 1783 end of the American Revolution (Tellman, 1996), followed by spread beyond the geography over which one of its progenitors (sorghum) is routinely cultivated -- which is continuing (McDonald et al., 2009).

Historically, plant genetic material was generally freely collected and shared-often from ‘centers of diversity’ in developing countries, and with archival in ‘gene banks’ in developed countries. Concerns that advances such as improved cultivars developed in one country may offer no benefit to the source of salient germplasm, have motivated a series of international legal frameworks designed to strike an appropriate balance between access to genetic and knowledge resources, and their utilization (Jefferson, 2025). This decades-long controversy shows little sign of resolution.

While international sharing of DNA mitigates some of these issues and sequencing costs are ever-more manageable, numerous additional considerations challenge research into plants and plant-associated organisms. Voluminous data exponentially increase computational load and can become a constraint, motivating the development of tools that take advantage of ongoing improvements in computation to filter quality genomics data addressing targeted questions from this ever growing ‘library’ of the history and diversity of life. As noted above, ‘reference’ genome quality is a moving standard, improving as technology empowers more precise and less costly sequencing. Relating DNA variation to trait variation requires more, and more rapid, phenotyping, using methods that need to be standardized and ideally automated. Funding constraints are a fact of life, and the indebtedness of many nations bode ill for near term improvements.

Beyond DNA exchange, two additional factors would potentially mitigate obstacles to progress. First, greater international scientific cooperation could minimize many constraints-there are some efforts such as joint funding programs between federal agencies in different countries, and philanthropic organizations willing to look beyond national boundaries-however regulatory and benefit sharing constraints remain deeply rooted.

Perhaps the single greatest change that might mitigate obstacles to research progress in plants and plant associated taxa, is a qualitative change in how society values ecosystem services. Once again, honey bee provides a striking example -- the value of pollination services they provide annually, in the United States alone, is variously estimated at 10–20 times the market value of the bees themselves, and an even higher multiple of the honey that most would consider the primary product of the bees. Even more difficult to reflect in efficient resource allocation are a host of other ‘Columbian Exchange’ crops, pests, weedy and invasive species with collective economic impact estimated at $26 billion annually (Crystal-Ornelas et al., 2021). One only hopes that greater appreciation of the value of ecosystem services, or the cost of neglecting them, will not require a crisis to which societal response is too little and too late.

In closing, genomics is no longer constrained in application to favorable models but can be readily integrated into research of non-specialists. For taxa of interest, entire genomes of individuals and even diversity panels sampling a gene pool are readily sequenced and annotated; gene expression either broadly or under specific conditions can be characterized in exquisite detail; and trait variation circumscribed to small genomic intervals; each of these goals leveraging an ever-richer body of knowledge from prior efforts in other taxa. Tremendous excitement accompanied the accomplishments of early milestones such as the sequencing of the first plant genome (Initiative, 2000), and a year later, the human genome-however, the true impact of genomics is only beginning to be felt, as pioneering of advances toward its use in biomedicine are leveraged into benefits across biology and evolution.

## Data Availability

The original contributions presented in the study are included in the article/supplementary material, further inquiries can be directed to the corresponding author.
